# Molecular characterization and functional analysis of a piscidin gene in large yellow croaker (*Larimichthys crocea*)

**DOI:** 10.13918/j.issn.2095-8137.2016.6.347

**Published:** 2016-11-18

**Authors:** Jing YANG, Xin-Jiang LU, Fang-Chao CHAI, Jiong CHEN

**Affiliations:** ^1^ Laboratory of Biochemistry and Molecular Biology, School of Marine Sciences, Ningbo University, Ningbo 315211, China; ^2^ Collaborative Innovation Center for Zhejiang Marine High-Efficiency and Healthy Aquaculture, Ningbo University, Ningbo 315211, China

**Keywords:** Antimicrobial activity, Large yellow croaker, Piscidin, Survival rate, *Vibrio alginolyticus*

## Abstract

The piscidin family, which includes potent antimicrobial peptides with broad-spectrum activity, plays an important role in the innate immune system of fish. In this study, we cloned piscidin-5-like type 3 (*Lcpis5lt3*) in large yellow croaker (*Larimichthys crocea*). Multiple alignments with other known piscidins revealed amino acid conservation throughout the fish, especially at the signal peptide (22 amino acids). The phylogenetic tree confirmed that Lcpis5lt3 and large yellow croaker piscidin-5-like proteins were grouped together to form a branch. Quantitative real-time PCR revealed that *Lcpis5lt3* was expressed in a wide range of tissues, including the brain, muscle, gill, head kidney, intestine, kidney, liver, and spleen. The highest mRNA expression level of *Lcpis5lt3* was found in the spleen. After *Vibrio alginolyticus* infection, mRNA expression was rapidly upregulated in the liver, head kidney, gill, kidney, and intestine at 4, 8, 12, and 24 h post infection (hpi), whereas there were no significant changes in the spleen. The antimicrobial spectrum showed that the synthetic mature peptide of Lcpis5lt3 exhibited different activity *in vitro* against various bacteria, such as *Aeromonas hydrophila*, *V. anguillarum*, *V. alginolyticus*, *V. parahaemolyticus*, *Staphylococcus aureus*, and *Listeria monocytogenes*. In addition, survival rates from the *in vivo* assay indicated that the synthetic peptide of Lcpis5lt3 increased the survival rate of large yellow croaker after *V*. *alginolyticus* challenge, resulting in a decline in bacterial burden and mRNA expression levels of interleukin-1β, interleukin-10, and tumor necrosis factor-α. These data suggest that Lcpis5lt3 plays an important role in innate immunity in large yellow croaker and might represent a potential therapeutic agent against pathogen invasion.

## INTRODUCTION

Large yellow croaker (*Larimichthys crocea*) is an economically important marine species of cultured fish (Niu et al., 2013). However, the aquaculture of large yellow croaker has suffered significant economic losses due to various infectious diseases caused by marine microorganisms such as *Vibrio alginolyticus* (Chen et al., 2003; Liu et al., 2016). *Vibrio alginolyticus* is an important pathogen that can cause disease in marine cultured fish (Samad et al., 2014). Infection leads to the upregulation of inflammatory cytokines, such as interleukin-1β (IL-1β), interleukin-10 (IL-10), and tumor necrosis factor-α (TNF-α) (Kayansamruaj et al., 2014; Ringø, 2011; Seppola et al., 2008). Infection also increases bacterial burden, which triggers multiple inflammatory mechanisms. Therefore, bacterial burden is an important indicator for innate host immunity in response to infection (Gomes et al., 2013). Increased lethality is also observed in various teleosts infected with pathogens (Chen et al., 2014; Li et al., 2014a). Nowadays, a variety of effective vaccines and medicines have been developed to control marine pathogens; however, these drugs often negatively affect the marine environment and fish themselves (Cabello et al., 2013). Therefore, there is an increasing demand for effective and environmentally friendly commercial therapeutics against marine microorganisms. In this respect, considerable attention has been paid to antimicrobial polypeptides (AMPs), known as endogenous antibiotics (Mukherjee & Hooper, 2015).

AMPs, a family of peptides and proteins with low molecular weight, are present in virtually all life forms (from prokaryotes to eukaryotic plants and animals) (Zasloff, 2002). These peptides are critical components of the innate immune system in low vertebrate hosts (Corrales et al., 2010; Lauth et al., 2002). Many AMPs have been identified from fish, including cathelicidin, histone-derived peptides, defensin, and hepcidin (Katzenback, 2015). Piscidin family proteins possess antimicrobial activity and include pleurocidin, moronecidin, chrysophsin, and dicentracin (Masso-Silva & Diamond, 2014; Umasuthan et al., 2016). In teleosts, piscidin genes have been cloned and reported in some species, including Atlantic cod (*Gadus morhua*) (Fernandes et al., 2010; Ruangsri et al., 2012), rock bream (*Oplegnathus fasciatus*) (Umasuthan et al., 2016), tilapia (*Oreochromis niloticus*) (Lin et al., 2016; Peng et al., 2012), hybrid striped bass (Noga et al., 2009; Salger et al., 2011; Silphaduang & Noga, 2001), and mandarin fish (*Siniperca chuatsi*) (Sun et al., 2007). The piscidin gene shares a common prepropeptide structure consisting of a signal peptide, a mature peptide, and a C-terminal prodomain of varied sequence and length (Lauth et al., 2002; Sun et al., 2007). As the major class of AMPs, piscidin displays potent broad-spectrum activity against bacteria (Silphaduang & Noga, 2001), fungi (Sung et al., 2008), parasites (Colorni et al., 2008), and even viruses (Chinchar et al., 2004). 

Recently, a piscidin-like antimicrobial peptide was isolated from large yellow croaker, and was determined to be a typical gill-expressed peptide distributed in various tissues (Niu et al., 2013). Furthermore, two types of *piscidin-5*-*like* sequences have been found in large yellow croaker, with their gene structure and sequence characteristics described (Zhou et al., 2014). The large yellow croaker *piscidin-5*-*like* gene and hybrid striped bass *piscidin-5* gene are reported to be most abundant in the head kidney and intestine, respectively (Salger et al., 2011; Zhou et al., 2014). The synthetic piscidin-4 peptide of hybrid striped bass shows antimicrobial activity against *Staphy lococcus aureus*, *Streptococcus iniae*, *Escherichia coli*, and *V. anguillarum* (Noga et al., 2009), and the synthetic piscidin-like peptide of large yellow croaker exhibits broad antimicrobial activity against *S. aureus*, *E. coli*, *Aspergillus niger*, and *Cryptocaryon irritans* in parasitic stages (Niu et al., 2013). These results show the high diversity of piscidin in mRNA expression and function in different fish. However, the effects of teleost piscidin on host defenses against pathogens *in vivo* are still unclear.

In this study, we characterized the cDNA sequence encoding a piscidin-like peptide, *Lcpis5lt3*, from large yellow croaker. Its mRNA expression in different tissues post *V. alginolyticus* infection was studied using quantitative PCR. In addition, the antimicrobial activity of synthetic peptides was also investigated *in vitro* and *in vivo*.

## MATERIALS AND METHODS

### Fish rearing

Healthy large yellow croaker, without pathological signs and weighing 35-40 g (fish age 7-9 months), were obtained from a commercial farm in Ningbo, China. Each 10 fish were kept in 100 L tanks at 25-27℃ in a recirculating system with filtered sea water. Detection was performed to ensure no bacteria were present in the seawater during the experiment. After acclimating for one week, the fish were used in the experiments described below. All experiments were approved by the Experimental Animal Management Law of China and the Animal Ethics Committee of Ningbo University. 

### Bacterial challenge

Overnight cultures of *V. alginolyticus* ATCC 17749 were diluted to 1: 100 in Tryptic Soy Broth Medium (TSB) (Sigma, Shanghai, China), grown at 28℃ with shaking, and harvested in the logarithmic phase of growth. The cells were washed, resuspended, and diluted to the appropriate concentration in sterile PBS. Sixteen fish were challenged by intraperitoneal injection with 5×10^6^ colony forming units (CFUs) of *V. alginolyticus* (in 100 μL PBS) per fish, and sixteen other fish were injected with 100 μL of PBS per fish as a negative control. The liver, spleen, head kidney, kidney, intestine, muscle, brain, and gill were collected from four fish at each time point at 4, 8, 12, and 24 h post-injection (hpi), as previously reported (Wu et al., 2015), then preserved at -70℃ until examination.

### Sequence analysis

Gene sequences used for multiple alignment and phylogenetic analysis are listed in Table 1. The similarity between the obtained sequences with other known sequences was analyzed using BLAST search (http://blast.ncbi.nlm.nih.gov/Blast.cgi). The cleavage site of signal peptides was predicted by the SignalP4.1 program (http://www.cbs.dtu.dk/services/SignalP/). Protein analysis was performed using online software on the ExPASy Server (http://www.expasy.org/tools/). Multiple sequence alignment was analyzed using the ClustalW program (http://clustalw.ddbj.nig.ac.jp/), and phylogenetic and molecular evolutionary analyses were conducted using MEGA version 5.0 (Tamura et al., 2011).

**1:** Piscidin sequences used for multiple sequence alignment and phylogenetic tree analysis

Species		Gene	GenBank ID
Latin name	English name		
*Larimichthys crocea*	Large yellow croaker	Piscidin5lt3	KX870851
*Larimichthys crocea*	Large yellow croaker	Piscidin5lt2	KJ879923
*Larimichthys crocea*	Large yellow croaker	Piscidin5l	KJ879922
*Larimichthys crocea*	Large yellow croaker	Piscidin l	EU741827
*Oplegnathus fasciatus*	Rock bream	Piscidin	AB703274
*Epinephelus malabaricus*	Malabar grouper	Piscidin1	JX412481
*Epinephelus malabaricus*	Malabar grouper	Piscidin2	JX412480
*Epinephelus coioides*	Orange spotted grouper	Piscidin	JQ823163
*Epinephelus coioides*	Orange spotted grouper	Piscidin l	EU741829
*Epinephelus bruneus*	Longtooth grouper	Piscidin l	JN216987
*Epinephelus bleekeri*	Duskytail grouper	Piscidin l	HQ437912
*Epinephelus fuscoguttatus*	Brown marbled grouper	Piscidin l	GU592793
*Epinephelus akaara*	Red spotted grouper	Piscidin l	EU741828
*Morone chrysops*	White bass	Piscidin1	AF394243
*Morone saxatilis*	Striped bass	Piscidin2	AF394244
*M. chrysops × M. saxatilis*	Hybrid striped bass	Piscidin4	HM596029
*M. chrysops × M. saxatilis*	Hybrid striped bass	Piscidin5	HM596030
*Dicentrarchus labrax*	European sea bass	Dicentracin	AY303949
*Siniperca chuatsi*	Mandarin fish	Moronecidin	AY647433

### Quantitative PCR (qPCR)

Changes in mRNA expression of *Lcpis5lt3* following *V. alginolyticus* infection were analyzed by qPCR, as previously described (Lu et al., 2016; Wu et al., 2015). Total RNA was extracted from large yellow croaker tissues using RNAiso reagents (TaKaRa). Gene-specific primers were designed based on the cloned cDNA fragments of Lcpis5lt3, LcIL-1β, LcTNF-α, and LcIL-10 (Table 2). Lcpis5lt3 and two types of piscidin-5-like gene sequences were subjected to nucleotide sequence alignment, and the primers of Lcpis5lt3 were designed in the ORF region of a 153-262 bp portion with low sequence identity. BLAST searching indicated that these primer sequences did not share sequence homology with any known large yellow croaker gene sequence, per the large yellow croaker genome (Wu et al., 2014). As an internal PCR control, primers 18S rRNA F and 18S rRNA R were used to amplify a 200-bp fragment of the housekeeping large yellow croaker 18S rRNA (Lc18S rRNA) gene (Accession No. JN211788.1) (Table 2). QPCR was conducted on an ABI StepOne Real-Time PCR System (Applied Biosystems, USA) using SYBR premix Ex Taq (Perfect Real Time) (TaKaRa) in accordance with the manufacturer's instructions. The reaction mixture was incubated for 300 s at 95℃, followed by 40 amplification cycles of 30 s at 95℃, 30 s at 60℃, and 30 s at 72℃. After amplification, melt curves were obtained by slow heating from 60℃ to 95℃ at 0.1℃/s, with continuous fluorescence collection, confirming that only our specific product peaks were detected. Fish were kept in eight aquaria. Two fish in each aquarium were biologically repeated twice, and the fish experiment was repeated once, for a total of four biological replicates. The mRNA expression of Lcpis5lt3 was normalized against that of 18S rRNA using the 2^-ΔΔCT^ method.

**2:** Oligonucleotide primers used in this work

Gene	Primer	Sequence (5'-3')	Amplification of length (bp)	Tm (℃)
Lcpis5lt3	Lcpis5lt3 F	ATTGTATCGATCTCGCTGGC	101	58
	Lcpis5lt3 R	CATAATTGGGTGGAAAACGG		55
Lc18S rRNA	Lc18S rRNA F	CTCTTAGCTGAGTGTCCCGC	200	60
	Lc18S rRNA R	ACCTCTAGCGGCACAATACG		60
LcIL-1β	LcIL-1β F	ATCTGGCAAGGATCAGCTCA	108	59
	LcIL-1β R	ACCAGTTGTTGTAGGGGACG		60
LcTNF-α	LcTNF-α F	TGGAGTGGAAGAACGGTCAA	173	59
	LcTNF-α R	GAGAGGTGTGAGGCGTTTCC		61
LcIL-10	LcIL-10 F	CAAGAGCATGAAGCCTCACA	169	58
	LcIL-10 R	GCCCACGGCCTTAAATAGAC		59

### Antimicrobial activity assays

The mature peptide of Lcpis5lt3 was chemically synthesized with over 95% purity (GL Biochem, Shanghai, China). The antimicrobial activity was determined against a panel of microorganisms. A micro-dilution assay was used to determine the minimal inhibitory concentration (MIC) of the various agents, as previously described with some modification (Li et al., 2014b). Inhibition was defined as growth lesser or equal to one-half of the growth observed in control wells where no peptide was added (Douglas et al., 2003). Briefly, serial dilutions of the peptides were made at 100, 50, 25, 12.5, 6.25, 3.125, and 1.563 μg/mL in 96-well microtiter plates. Each well contained 100 μL of a bacterial cell suspension at 1×10^5^ CFU/mL and 11 μL of test peptide. After incubating for 24 h at the appropriate temperature, microbial growth was examined. All tests were performed in triplicate and each individual experiment was replicated in quadruplicate. For each series of experiments, PBS was employed as a negative control.

### Fish survival assay

Fish were divided into three groups (each containing 16 fish) for survival assay. Fish were injected intraperitoneally (ip) with 5×10^6^ CFU/g *V. alginolyticus*. After 30 min, fish received ip injections of 1.0 μg/g Lcpis5lt3 or 0.1 μg/g Lcpis5lt3 of fish weight, while the control group received PBS 30 min post injection. Fish were observed every 24 h for death or moribund state for 8 d.

### Bacterial burden in tissues

Three groups, each containing six fish, were ip-injected with *V. alginolyticus* (5×10^6^ CFU/g). At 30 min post-infection, the fish received ip injections of different doses of Lcpis5lt3 or PBS, respectively. Fish were sacrificed 12 h after ip injection, and the liver, kidney, spleen, and blood were collected. The tissues from each large yellow croaker were weighed and homogenized in 1 mL of sterile PBS (pH 7.2). Homogenates and blood were serially diluted in sterile PBS (pH 7.2) and then plated onto separate Thiosulfate Citrate Bile Salts (TCBS) agar plates for 18 h at 28℃. CFUs were then calculated in all plates and multiplied by the dilution factor. Tissue samples were normalized to tissue weight (0.1 g), and blood samples were normalized to blood volume (0.1 mL).

### Statistical analysis

All data were described as mean±SEM. Statistical analysis of results was conducted by one-way analysis of variance (ANOVA) with SPSS version 13.0 (SPSS Inc, Chicago, USA). *P*-values of less than 0.05 were considered statistically significant.

## RESULTS

### *Lcpis5lt3* gene analysis

**Figure 1:**
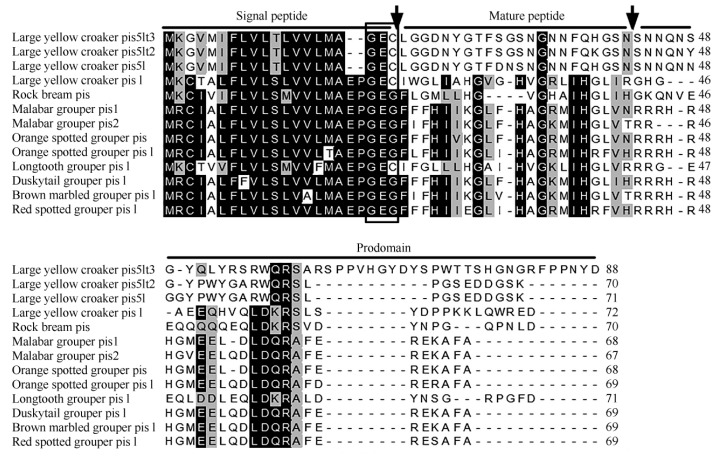
Multiple alignment of the predicted Lcpis5lt3 amino acid sequence with other known piscidins

Using the liver transcriptome analysis of large yellow croaker, the cDNA sequence of the *Lcpis5lt3* gene was identified by BLAST search and submitted to the DDBJ/EMBL/GenBank databases under accession number KX870851. Computer analysis showed that the large yellow croaker *pis5lt3*, *piscidin-5*-*like*, and *piscidin-5*-*like type 2* cDNA sequences contained open reading frames (ORFs) of 264 bp, 213 bp, and 210 bp that encoded an 88, 71, and 70 amino acid peptide, respectively. The peptide of Lcpis5lt3 had an estimated molecular weight (MW) of 9.78 kDa and theoretical isoelectric point (pI) of 8.93. All piscidin-5s were comprised of an N-terminal signal peptide (22 amino acids), a mature peptide (22 amino acids), and a C-terminal prodomain (Figure 1). The deduced cleavage site for the signal peptide was between positions 22 and 23 (GEC-LG), like that of most piscidin sequences referenced, terminating at the motif GEC, GES, or GEG (Figure 1) (Douglas et al., 2003). However, the peptide length of Lcpis5lt3 was longer than that of piscidin-5-like type 2 and piscidin-5-like.

**Figure 2:**
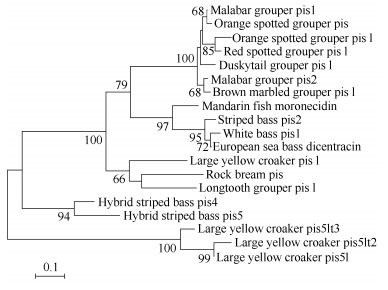
Phylogenetic (neighbor-joining) analysis of the complete amino acid sequences of a piscidin protein using the MEGA5.0 program

Amino acid sequence alignment of Lcpis5lt3 with closely related sequences revealed conservation in the signal peptide region (Figure 1), but low similarity in the mature peptide and prodomain. In general, Lcpis5lt3 showed low identity (less than 62.5%) to other known piscidin sequences. Based on the known fish piscidin amino acid sequences, a phylogenetic tree was constructed using the neighbor-joining method (Figure 2). Results showed that Lcpis5lt3 and other large yellow croaker piscidin-5s grouped together to form a large yellow croaker piscidin-5 cluster.

### Antimicrobial spectrum

The antibacterial activity of the synthesized mature peptide was determined against a panel of microorganisms using the MIC method. The MIC values obtained are reported in Table 3. The synthesized mature peptide of Lcpis5lt3 exhibited activity against *Aeromonas hydrophila*, *V. anguillarum*, *and V. alginolyticus* at 100 μg/mL. It also displayed antibacterial activity with MICs at 50 μg/mL and 6.25 μg/mL against *V. parahaemolyticus* and *S. aureus*, respectively. Lcpis5lt3 had antibacterial activity against *Listeria monocytogenes* with MIC at 3.125 μg/mL. However, this peptide had no effect on *Edwardsiella tarda*, *V. vulnificus*, *V. harveyi*, or *S. iniae* at the concentration tested.

**3:** Antimicrobial activity of synthetic Lcpis5lt3

Bacteria	Strains	Culture medium	Culture temperature (℃)	Lcpis5lt3 MIC (μg/mL)
*Edwardsiella tarda*	Et-CD	LB	37	-
*Aeromonas hydrophila*	ATCC7966	LB	37	100
*Staphylococcus aureus*	ATCC6538	LB	37	6.25
*Listeria monocytogenes*	ATCC19115	BHI	37	3.125
*Vibrio anguillarum*	ATCC19264	TSB	28	100
*Vibrio alginolyticus*	ATCC17749	TSB	28	100
*Vibrio vulnificus*	ATCC27562	TSB	28	-
*Vibrio parahaemolyticus*	ATCC33847	TSB	28	50
*Vibrio harveyi*	ATCC33866	TSB	28	-
*Streptococcus iniae*	ATCC29178	BHI	37	-

"-" Means no inhibition found at 100 μg/mL.

### Constitutive and induced expression in different tissues

**Figure 3:**
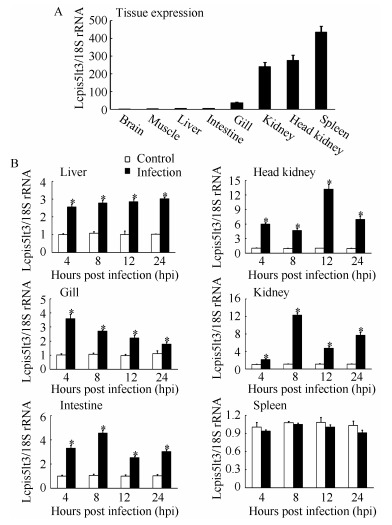
QPCR analysis of Lcpis5lt3 mRNA expression in different tissues

QPCR was performed to analyze the temporal expression profile of *Lcpis5lt3* in different tissues of healthy large yellow croaker. The results showed that *Lcpis5lt3* exhibited constitutive expression in all examined tissue, including brain, muscle, liver, intestine, gill, kidney, head kidney, and spleen. The highest expression level of *Lcpis5lt3* was detected in the spleen, followed by the head kidney and kidney (Figure 3A). After *V. alginolyticus* infection, the mRNA expression of *Lcpis5lt3* was rapidly upregulated in liver, head kidney, gill, kidney, and intestine at 4, 8, 12, and 24 hpi, whereas no significant changes were found in the spleen at 4, 8, 12, and 24 hpi (Figure 3B).

### Effect of Lcpis5lt3 on the survival rate of *V. alginolyticus*-infected fish

**Figure 4:**
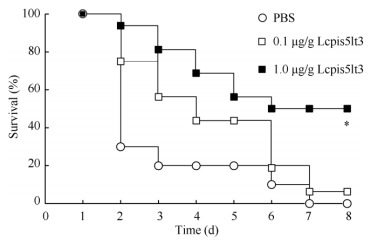
Effect of Lcpis5lt3 on the survival rate of large yellow croaker

We investigated the bactericidal effects of synthesized mature peptide *in vivo* by monitoring the survival of large yellow croaker infected with *V. alginolyticus* prior to treatment with different concentrations of Lcpis5lt3. All PBS-treated large yellow croaker infected with *V. alginolyticus* died within 7 d after infection. Large yellow croaker treatment with Lcpis5lt3 decreased the mortality rate (Figure 4). At 8 d after *V. alginolyticus* infection, the survival rates were 6% and 50% for large yellow croaker treated with 0.1 μg/g and 1.0 μg/g Lcpis5lt3, respectively (Figure 4).

### Bacterial burden in tissues and blood

**Figure 5:**
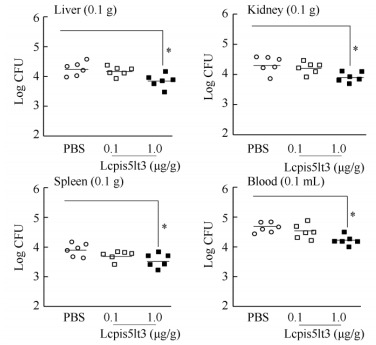
Effect of Lcpis5lt3 on bacterial burden in large yellow croaker liver, spleen, kidney, and blood

To examine the impact of the synthesized mature peptide of Lcpis5lt3 on bacterial proliferation and dissemination *in vivo*, the bacterial loads were quantitated in the liver, spleen, kidney, and blood following ip-injection with 0.1 μg/g Lcpis5lt3 or 1.0 μg/g Lcpis5lt3 in *V. alginolyticus*-challenged fish. Fish treated with 0.1 μg/g Lcpis5lt3 and 1.0 μg/g Lcpis5lt3 all showed a reduction in *V. alginolyticus* load in the liver, spleen, kidney, and blood 12 hpi after *V. alginolyticus* challenge in comparison with the PBS-treated control group (Figure 5). There were significant differences between the control and 1.0 μg/g Lcpis5lt3 groups in all tested tissues, whereas the 0.1 μg/g Lcpis5lt3-treated group showed only small variation (Figure 5).

### Effect of Lcpis5lt3 on cytokine expression following infection

**Figure 6:**
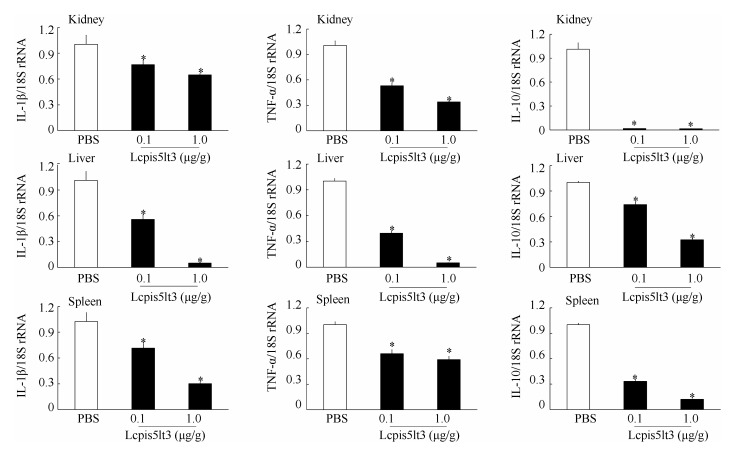
Effect of Lcpis5lt3 on mRNA levels of LcTNF-α, LcIL-1β, and LcIL-10

To explore the effect of Lcpis5lt3 on inflammatory gene expression *in vivo*, the mRNA levels for inflammatory cytokines LcTNF-α, LcIL-1β, and LcIL-10 were evaluated in tissues collected from the fish after Lcpis5lt3 treatment and PBS-treated controls following infection with *V. alginolyticus*. QPCR analysis revealed a significant decrease in the expressions of LcTNF-α, LcIL-1β, and LcIL-10 transcripts in the tissues of fish treated with 0.1 μg/g or 1.0 μg/g Lcpis5lt3 compared with fish treated with PBS (Figure 6).

## DISCUSSION

In the present study, *Lcpis5lt3* was identified and characterized in large yellow croaker. Lcpis5lt3 comprised a signal peptide, mature peptide, and prodomain, as shared by other piscidin paralogues (Buonocore et al., 2012; Niu et al., 2013). Similar to other piscidins (Zhou et al., 2014), Lcpis5lt3 showed more conservation in the signal peptide and less conservation in the mature peptide and prodomain. The phylogenetic tree confirmed that Lcpis5lt3 was grouped together with large yellow croaker piscidin-5-like type 2 and piscidin-5-like to form a cluster. This result revealed that large yellow croaker pis5lt3 was a variation type of the piscidin-5-like peptide. According to previous research, several piscidin paralogues can be found in a single fish species. For example, piscidin-4 and piscidin-5 genes are found in hybrid striped bass (Salger et al., 2011); piscidin-1, piscidin-2, piscidin-3, piscidin-4, and piscidin-5 genes are found in tilapia (Peng et al., 2012); and piscidin-1, piscidin-2, and piscidin-2-β genes are found in Atlantic cod (Ruangsri et al., 2012). There are also several piscidin genes found in large yellow croaker, such as piscidin-like, piscidin-5-like, and piscidin-5-like type 2 (Zhou et al., 2014). These results show a high diversity of piscidin in different fish.

The transcripts of piscidin genes are widely distributed in various tissues (Buonocore et al., 2012; Salger et al., 2011). In rock bream, the piscidin gene is highly expressed in the gills of healthy fish (Bae et al., 2016). In tilapia, piscidin-2 is abundant in the skin, head kidney, and spleen; piscidin-3 is abundant in the skin, head kidney, and gill; and piscidin-4 is abundant in the intestine (Peng et al., 2012). In the large yellow croaker, the piscidin-like gene is most abundantly expressed in the gill of unchallenged fish (Niu et al., 2013), and the piscidin-5-like is most abundant in the head kidney (Zhou et al., 2014). In this study, the *Lcpis5lt3* transcript was highly expressed in the spleen, head kidney, and kidney of the large yellow croaker, suggesting that piscidin genes have a variety of tissue expression patterns. In mandarin fish, piscidin gene mRNA expression is upregulated in the intestine, spleen, kidney, liver, skin, and gill after stimulation with LPS (Sun et al., 2007). In large yellow croaker, piscidin-like gene mRNA expression is significantly upregulated in the gill, skin, spleen, head kidney, liver, and intestine after *C. irritans* infection (Niu et al., 2013). In the current study, the mRNA expression levels of *Lcpis5lt3* were upregulated in the liver, head kidney, gill, kidney, and intestine after *V. alginolyticus* infection. These results suggest that most fish piscidin in a variety of tissues were upregulated after infection. Furthermore, we found there were no significant changes in *Lcpis5lt3* in the spleen after infection. However, this mechanism needs further investigation. 

Unique for the field of fish research, AMPs have potential applications to prevent pathogenic microbes in aquaculture (Masso-Silva & Diamond, 2014). The constant risk of large-scale microbial infection that can lead to significant economic losses requires new strategies to prevent or treat these pathogens (Masso-Silva & Diamond, 2014). In rock bream, the synthetic piscidin peptide exhibits antimicrobial activity against *E. tarda*, *V. vuln ificus*, *V. harveyi*, and *S. iniae* (Bae et al., 2016). In tilapia, five synthetic piscidin peptides have been shown to exhibit antimicrobial activity against *V. vulnificus*, *V. alginolyticus*, *A. hydrophila*, and *Pseudomonas aeruginosa* (Peng et al., 2012). In contrast, our results showed that Lcpis5lt3 had no effect on *E. tarda*, *V. vulnificus*, *V. harveyi*, or *S. iniae* at the antimicrobial activity tested. However, Lcpis5lt3 exhibited antimicrobial activity against *L. monocytogenes*, *S. aureus*, *V. anguillarum*, *V. alginolyticus*, *V. parahaemolyticus*, and *A. hydrophila in vitro*. These results demonstrate that Lcpis5lt3 possesses broad-spectrum antimicrobial activity, and different piscidins in teleosts possess spectrum variety in antimicrobial activity. 

To evaluate the antimicrobial activity of Lcpis5lt3 against *V. alginolyticus in vivo*, we performed the survival rate assay. Our results showed that the synthesized mature peptide of Lcpis5lt3 can increase the survival rate of fish infected with *V. alginolyticus* at different concentrations. The overall survival rate of the 1.0 μg/g Lcpis5lt3 group was higher than that of the 0.1 μg/g Lcpis5lt3 group after infection; this effect was accompanied by a lower bacterial burden and a decline in LcTNF-α, LcIL-1β, and LcIL-10 mRNA expression. The cytokines TNF-α and IL-1β in teleosts are powerful proinflammatory cytokines released by several immune cells during infection or tissue damage and are involved in a diverse range of inflammatory and infectious conditions (Roca et al., 2008; Seppola et al., 2008; Wu et al., 2015). However, IL-10 is a critical anti-inflammatory cytokine, whose expression is induced after proinflammatory mediators. It helps control immune responses and thereby minimize tissue damage (Secombes et al., 2011; Wang and Secombes, 2013; Zhu et al., 2013). As an autoregulatory mediator, IL-10 has important regulatory effects on immunological and inflammatory responses due to its capacity to inhibit the production of proinflammatory cytokines by monocytes (Ringø, 2011). In this study, we found that LcTNF-α, LcIL-1β, and LcIL-10 mRNA expression were all downregulated in the 0.1μg/g and 1.0 μg/g Lcpis5lt3 groups after infection, suggesting that inflammation in fish after *V.alginolyticus* infection was relieved. Similar results were found with tilapia piscidin-4 (TP4). For example, mice treated with TP4 resulted in the downregulation of TNF-α, IL-1β, and IL-10 (Narayana et al., 2015). However, it is still unclear whether piscidins can directly downregulate inflammatory cytokines, such as the AMPS of other fish (Chen et al., 2016; Li et al., 2015), or only kill pathogens to reduce the expression of inflammatory cytokines. Further investigation is needed to elucidate the role of Lcpis5lt3 in the immune responses of large yellow croaker after infection. 

In conclusion, we characterized *Lcpis5lt3* as a member of the piscidin family in large yellow croaker. Results showed that *V. alginolyticus* infection led to the alteration of *Lcpis5lt3* mRNA expression in different tissues. Antimicrobial assays *in vitro* and *in vivo* also showed that the synthetic mature peptide had broad spectrum antimicrobial activity *in vitro* and increased fish survival upon bacterial infection *in vivo*. These data provide new insights into the innate immunity of large yellow croaker against pathogens and reveal the value of piscidin as a therapeutic agent to control microbial infections.

## References

[1] Bae JS, Jung JM, An CM, Kim JW, Hwang SD, Kwon MG, Park MA, Kim MC, Park CI. 2016 Piscidin: antimicrobial peptide of rock bream, *Oplegnathus fasciatus*. *Fish & Shellfish Immunology*, 51 136- 142. 26876358 10.1016/j.fsi.2016.02.010

[2] Buonocore F, Randelli E, Casani D, Picchietti S, Belardinelli MC, de Pascale D, De Santi C, Scapigliati G. 2012 A piscidin-like antimicrobial peptide from the icefish *Chionodraco hamatus* (Perciformes: Channichthyidae): molecular characterization, localization and bactericidal activity. *Fish & Shellfish Immunology*, 33 (5): 1183- 1191. 22982327 10.1016/j.fsi.2012.09.005

[3] Cabello FC, Godfrey HP, Tomova A, Ivanova L, Dölz H, Millanao A, Buschmann AH. 2013 Antimicrobial use in aquaculture re-examined: its relevance to antimicrobial resistance and to animal and human health. *Environmental Microbiology*, 15 (7): 1917- 1942. 23711078 10.1111/1462-2920.12134

[4] Chen J, Chen Q, Lu XJ, Li CH. 2014 LECT2 improves the outcomes in ayu with *Vibrio anguillarum* infection via monocytes/macrophages. *Fish & Shellfish Immunology*, 41 (2): 586- 592. 25462453 10.1016/j.fsi.2014.10.012

[5] Chen J, Chen Q, Lu XJ, Chen J. 2016 The protection effect of LEAP-2 on the mudskipper (*Boleophthalmus pectinirostris*) against *Edwardsiella tarda* infection is associated with its immunomodulatory activity on monocytes/macrophages. *Fish & Shellfish Immunology*, 59 66- 76. 27765699 10.1016/j.fsi.2016.10.028

[6] Chen XH, Lin KB, Wang XW. 2003 Outbreaks of an iridovirus disease in maricultured large yellow croaker, *Larimichthys crocea* (Richardson), in China. *Journal of Fish Diseases*, 26 (10): 615- 619. 14653319 10.1046/j.1365-2761.2003.00494.x

[7] Chinchar VG, Bryan L, Silphadaung U, Noga E, Wade D, Rollins-Smith L. 2004 Inactivation of viruses infecting ectothermic animals by amphibian and piscine antimicrobial peptides. *Virology*, 323 (2): 268- 275. 15193922 10.1016/j.virol.2004.02.029

[8] Colorni A, Ullal A, Heinisch G, Noga EJ. 2008 Activity of the antimicrobial polypeptide piscidin 2 against fish ectoparasites. *Journal of Fish Diseases*, 31 (6): 423- 432. 18471098 10.1111/j.1365-2761.2008.00922.x

[9] Corrales J, Mulero I, Mulero V, Noga EJ. 2010 Detection of antimicrobial peptides related to piscidin 4 in important aquacultured fish. *Developmental & Comparative Immunology*, 34 (3): 331- 343. 19913049 10.1016/j.dci.2009.11.004

[10] Douglas SE, Patrzykat A, Pytyck J, Gallant JW. 2003 Identification, structure and differential expression of novel pleurocidins clustered on the genome of the winter flounder, *Pseudopleuronectes americanus* (Walbaum). *European Journal of Biochemistry*, 270 (18): 3720- 3730. 12950255 10.1046/j.1432-1033.2003.03758.x

[11] Fernandes JMO, Ruangsri J, Kiron V. 2010 Atlantic cod piscidin and its diversification through positive selection. *PLoS One*, 5 (3): e9501- 20209162 10.1371/journal.pone.0009501 PMC2830478

[12] Gomes RN, Teixeira-Cunha MGA, Figueiredo RT, Almeida PE, Alves SC, Bozza PT, Bozza FA, Bozza MT, Zimmerman GA, Castro-Faria-Neto HC. 2013 Bacterial clearance in septic mice is modulated by MCP-1/CCL2 and nitric oxide. *Shock*, 39 (1): 63- 69. 23247123 10.1097/SHK.0b013e31827802b5 PMC3592381

[13] KATZENBACK BA. 2015 Antimicrobial peptides as mediators of innate immunity in teleosts. *Biology*, 4 (4): 607- 639. 26426065 10.3390/biology4040607 PMC4690011

[14] Kayansamruaj P, Pirarat N, Hirono I, Rodkhum C. 2014 Increasing of temperature induces pathogenicity of Streptococcus agalactiae and the up-regulation of inflammatory related genes in infected Nile tilapia (*Oreochromis niloticus*). *Veterinary Microbiology*, 172 (1-2): 265- 271. 24856132 10.1016/j.vetmic.2014.04.013

[15] Lauth X, Shike H, Burns JC, Westerman ME, Ostland VE, Carlberg JM, Van Olst JC, Nizet V, Taylor SW, Shimizu C, Bulet P. 2002 Discovery and characterization of two isoforms of moronecidin, a novel antimicrobial peptide from hybrid striped bass. *Journal of Biological Chemistry*, 277 (7): 5030- 5039. 11739390 10.1074/jbc.M109173200

[16] Li CH, Lu XJ, Li DF, Chen J. 2014a. Passive protective effect of chicken egg yolk immunoglobulins against experimental *Vibrio anguillarum* infection in ayu (*Plecoglossus altivelis*). *Fish & Shellfish Immunology*, 37 (1): 108- 114. 24486630 10.1016/j.fsi.2014.01.018

[17] Li CH, Lu XJ, Li MY, Chen J. 2015 Cathelicidin modulates the function of monocytes/macrophages via the P2X7 receptor in a teleost, *Plecoglossus altivelis*. *Fish & Shellfish Immunology*, 47 (2): 878- 885. 26525517 10.1016/j.fsi.2015.10.031

[18] Li HX, Lu XJ, Li CH, Chen J. 2014b. Molecular characterization and functional analysis of two distinct liver-expressed antimicrobial peptide 2 (LEAP-2) genes in large yellow croaker (*Larimichthys crocea*). *Fish & Shellfish Immunology*, 38 (2): 330- 339. 24727197 10.1016/j.fsi.2014.04.004

[19] Lin WC, Chang HY, Chen JY. 2016 Electrotransfer of the tilapia piscidin 3 and tilapia piscidin 4 genes into skeletal muscle enhances the antibacterial and immunomodulatory functions of Oreochromis niloticus. Fish & Shellfish Immunology, 50 200- 209. 26828260 10.1016/j.fsi.2016.01.034

[20] Liu L, Ge MF, Zheng XY, Tao Z, Zhou SM, Wang GL. 2016 Investigation of *Vibrio alginolyticus*, V. harveyi, and *V. parahaemolyticus* in large yellow croaker, *Pseudosciaena crocea* (Richardson) reared in Xiangshan Bay, China. *Aquaculture Reports*, 3 220- 224.

[21] Lu XJ, Zhang H, Yang GJ, Li MY, Chen J. 2016 Comparative transcriptome analysis on the alteration of gene expression in ayu (*Plecoglossus altivelis*) larvae associated with salinity change. *Zoological Research*, 37 (3): 126- 135. 27265650 10.13918/j.issn.2095-8137.2016.3.126 PMC4914575

[22] Masso-Silva JA, Diamond G. 2014 Antimicrobial peptides from fish. *Pharmaceuticals*, 7 (3): 265- 310. 24594555 10.3390/ph7030265 PMC3978493

[23] Mukherjee S, Hooper L V. 2015 Antimicrobial defense of the intestine. *Immunity*, 42 (1): 28- 39. 25607457 10.1016/j.immuni.2014.12.028

[24] Narayana JL, Huang HN, Wu CJ, Chen JY. 2015 Efficacy of the antimicrobial peptide TP4 against *Helicobacter pylori* infection: *in vitro* membrane perturbation *via* micellization and *in vivo* suppression of host immune responses in a mouse model. *Oncotarget*, 6 (15): 12936- 12954. 26002554 10.18632/oncotarget.4101 PMC4536990

[25] Niu SF, Jin Y, Xu X, Qiao Y, Wu Y, Mao Y, Su YQ, Wang J. 2013 Characterization of a novel piscidin-like antimicrobial peptide from *Pseudosciaena crocea* and its immune response to *Cryptocaryon irritans*. *Fish & Shellfish Immunology*, 35 (2): 513- 524. 23727503 10.1016/j.fsi.2013.05.007

[26] Noga EJ, Silphaduang U, Park NG, Seo JK, Stephenson J, Kozlowicz S. 2009 Piscidin 4, a novel member of the piscidin family of antimicrobial peptides. *Comparative Biochemistry and Physiology Part B: Biochemistry and Molecular Biology*, 152 (4): 299- 305. 10.1016/j.cbpb.2008.12.018 19266617

[27] Peng KC, Lee SH, Hour AL, Pan CY, Lee LH, Chen JY. 2012 Five different piscidins from Nile tilapia, *Oreochromis niloticus*: analysis of their expressions and biological functions. *PLoS One*, 7 (11): e50263- 23226256 10.1371/journal.pone.0050263 PMC3511469

[28] Ringø E. 2011 Evaluation of probiotic strain *Bacillus subtilis* C-3102 as a feed supplement for koi carp (*Cyprinus carpio*). *Journal of Aquaculture Research & Development*, S1 005-

[29] Roca FJ, Mulero I, López-Muñoz A, Sepulcre MP, Renshaw SA, Meseguer J, Mulero V. 2008 Evolution of the inflammatory response in vertebrates: fish TNF-α is a powerful activator of endothelial cells but hardly activates phagocytes. *The Journal of Immunology*, 181 (7): 5071- 5081. 18802111 10.4049/jimmunol.181.7.5071

[30] Ruangsri J, Salger SA, Caipang CMA, Kiron V, Fernandes JMO. 2012 Differential expression and biological activity of two piscidin paralogues and a novel splice variant in Atlantic cod (*Gadus morhua* L.). *Fish & Shellfish Immunology*, 32 (3): 396- 406. 22178249 10.1016/j.fsi.2011.11.022

[31] Salger SA, Reading BJ, Baltzegar DA, Sullivan CV, Noga EJ. 2011 Molecular characterization of two isoforms of piscidin 4 from the hybrid striped bass (*Morone chrysops × Morone saxatilis*). *Fish & Shellfish Immunology*, 30 (1): 420- 424. 20955800 10.1016/j.fsi.2010.10.009

[32] Samad APA, Santoso U, Lee MC, Nan FH. 2014 Effects of dietary katuk (*Sauropus androgynus* L. Merr.) on growth, non-specific immune and diseases resistance against *Vibrio alginolyticus* infection in grouper *Epinephelus coioides*. *Fish & Shellfish Immunology*, 36 (2): 582- 589. 24296304 10.1016/j.fsi.2013.11.011

[33] Secombes CJ, Wang T, Bird S. 2011 The interleukins of fish. *Developmental & Comparative Immunology*, 35 (12): 1336- 1345. 21605591 10.1016/j.dci.2011.05.001

[34] Seppola M, Larsen AN, Steiro K, Robertsen B, Jensen I. 2008 Characterisation and expression analysis of the interleukin genes, IL-1β, IL-8 and IL-10, in Atlantic cod (*Gadus morhua* L.). *Molecular Immunology*, 45 (4): 887- 897. 17875325 10.1016/j.molimm.2007.08.003

[35] Silphaduang U, Noga EJ. 2001 Peptide antibiotics in mast cells of fish. *Nature*, 414 (6861): 268- 269. 11713517 10.1038/35104690

[36] Sun BJ, Xie HX, Song Y, Nie P. 2007 Gene structure of an antimicrobial peptide from mandarin fish, *Siniperca chuatsi* (Basilewsky), suggests that moronecidins and pleurocidins belong in one family: the piscidins. *Journal of Fish Diseases*, 30 (6): 335- 343. 17498177 10.1111/j.1365-2761.2007.00789.x

[37] Sung WS, Lee J, Lee DG. 2008 Fungicidal effect and the mode of action of piscidin 2 derived from hybrid striped bass. *Biochemical and Biophysical Research Communications*, 371 (3): 551- 555. 18445475 10.1016/j.bbrc.2008.04.107

[38] Tamura K, Peterson D, Peterson N, Stecher G, Nei M, Kumar S. 2011 MEGA5: molecular evolutionary genetics analysis using maximum likelihood, evolutionary distance, and maximum parsimony methods. *Molecular Biology and Evolution*, 28 (10): 2731- 2739. 21546353 10.1093/molbev/msr121 PMC3203626

[39] Umasuthan N, Mothishri MS, Thulasitha WS, Nam BH, Lee J. 2016 Molecular, genomic, and expressional delineation of a piscidin from rock bream (*Oplegnathus fasciatus*) with evidence for the potent antimicrobial activities of Of-Pis1 peptide. *Fish & Shellfish Immunology*, 48 154- 168. 26549174 10.1016/j.fsi.2015.11.005

[40] Wang TH, Secombes CJ. 2013 The cytokine networks of adaptive immunity in fish. *Fish & Shellfish Immunology*, 35 (6): 1703- 1718. 24036335 10.1016/j.fsi.2013.08.030

[41] Wu CW, Zhang D, Kan MY, Lv ZM, Zhu AY, Su YQ, Zhou DZ, Zhang JS, Zhang Z, Xu MY, Jiang LH, Guo BY, Wang T, Chi CF, Mao Y, Zhou JJ, Yu XX, Wang HL, Weng XL, Jin JG, Ye JY, He L, Liu Y. 2014 The draft genome of the large yellow croaker reveals well-developed innate immunity. *Nature Communications*, 5 5227- 10.1038/ncomms6227 PMC4263168 25407894

[42] Wu J, Shi YH, Zhang XH, Li CH, Li MY, Chen J. 2015 Molecular characterization of an IL-1β gene from the large yellow croaker (*Larimichthys crocea*) and its effect on fish defense against *Vibrio alginolyticus* infection. *Zoological Research*, 36 (3): 133- 141. 26018856 PMC4790688

[43] Zasloff M. 2002 Antimicrobial peptides of multicellular organisms. *Nature*, 415 (6870): 389- 395. 11807545 10.1038/415389a

[44] Zhou QJ, Su YQ, Niu SF, Liu M, Qiao Y, Wang J. 2014 Discovery and molecular cloning of piscidin-5-like gene from the large yellow croaker (*Larimichthys crocea*). *Fish & Shellfish Immunology*, 41 (2): 417- 420. 25263194 10.1016/j.fsi.2014.09.023

[45] Zhu LY, Nie L, Zhu G, Xiang LX, Shao JZ. 2013 Advances in research of fish immune-relevant genes: a comparative overview of innate and adaptive immunity in teleosts. *Developmental & Comparative Immunology*, 39 (1-2): 39- 62. 22504163 10.1016/j.dci.2012.04.001

